# Phylogeny of Yellow Fever Virus, Uganda, 2016

**DOI:** 10.3201/eid2408.180588

**Published:** 2018-08

**Authors:** Holly R. Hughes, John Kayiwa, Eric C. Mossel, Julius Lutwama, J. Erin Staples, Amy J. Lambert

**Affiliations:** Centers for Disease Control and Prevention, Fort Collins, Colorado, USA (H.R. Hughes, E.C. Mossel, J.E. Staples, A.J. Lambert);; Uganda Virus Research Institute, Entebbe, Uganda (J. Kayiwa, J. Lutwama)

**Keywords:** *Suggested citation for this article*: Hughes HR, Kayiwa J, Mossel EC, Lutwama J, Staples JE, Lambert AJ. Phylogeny of yellow fever virus, Uganda, 2016. Emerg Infect Dis. 2018 Aug [*date cited*]. https://doi.org/10.3201/eid2408.180588

## Abstract

In April 2016, a yellow fever outbreak was detected in Uganda. Removal of contaminating ribosomal RNA in a clinical sample improved the sensitivity of next-generation sequencing. Molecular analyses determined the Uganda yellow fever outbreak was distinct from the concurrent yellow fever outbreak in Angola, improving our understanding of yellow fever epidemiology.

Yellow fever virus (YFV) remains a public health threat; outbreaks occur frequently in regions of Africa and South America to which it is endemic. Genetic analyses have identified 5 YFV genotypes circulating in Africa in distinct geographic regions (*1*,*2*). This information can be used to identify the origin of outbreaks.

In December 2015, a yellow fever outbreak was identified in Luanda, Angola (*3*). A rapid increase in the number of cases was observed in January 2016, and the outbreak subsequently spread to other areas of Angola and neighboring Democratic Republic of the Congo (*4*). In April 2016, yellow fever was identified in the southwestern district, Masaka, of Uganda (*4*). By June 2016, the Ministry of Health of Uganda had reported 68 suspected yellow fever cases, of which 3 probable and 7 confirmed cases were in the Masaka, Rukungiri, and Kalangala districts (*5*). The Uganda Virus Research Institute (Entebbe, Uganda) collaborated with the Centers for Disease Control and Prevention (CDC, Fort Collins, CO, USA) to confirm the presence of YFV RNA in human clinical samples and determine the molecular epidemiology of virus causing the Uganda outbreak.

Serum specimens from the Uganda 2016 outbreak were determined to be YFV RNA-positive by real-time reverse transcription PCR at the Uganda Virus Research Institute, and CDC confirmed the results using a previously published method (*6*). One serum sample was selected as the most viable candidate for next-generation sequencing because of its relative concentration of viral RNA, as determined by real-time reverse transcription PCR (cycle threshold <30). The sample was prepared for sequencing on the Ion Torrent Personal Genomics Machine system (Life Technologies, Carlsbad, CA, USA), as previously described (*7*). Initial sequencing did not result in any sequence reads aligning with a YFV reference template (SeqMan NGen; DNASTAR, Madison, WI, USA), suggesting that the YFV RNA in the sample was of low quality and/or quantity.

To enhance sequence coverage, we subjected RNA extracted from the selected serum sample to a targeted RNase-H (Epicentre, Madison, WI, USA) digestion to remove the contaminating carrier and ribosomal RNA, as previously described (*8*). Then we prepared a standard cDNA library and conducted Ion Torrent sequencing. Fastq files were again aligned to a YFV reference template in SeqMan NGen (DNASTAR). Targeted RNase-H treatment of the RNA sample resulted in 37,637 sequencing reads aligning to the reference template or 1.2% of all sequencing reads corresponding to 38% coverage of the complete YFV genome. Contigs representing partial sequences of 7 coding regions (capsid, membrane, envelope, nonstructural [NS] 1, NS2B, NS3, and NS5) of the 10 YFV genes were identified. The longest contigs and deepest coverage were identified in partial coding regions of the envelope (693 nt; GenBank accession no. MG757496), NS3 (963 nt; GenBank accession no. MG757497), and NS5 (450 nt; GenBank accession no. MG757498), which were subjected to BLAST analyses (https://blast.ncbi.nlm.nih.gov/Blast.cgi). The YFV Uganda 2016 strain envelope sequence was aligned with reference YFV genomes by using MAFFT through the EMBL-EBI server (http://www.ebi.ac.uk), and phylogenies were generated with BEAST 1.8.4 (*9*), as previously described (*7*).

BLAST analyses determined that the highest percentage identity (95%) is shared between the Uganda 2016 strain and strains from South Sudan 2003 in the envelope region (the only region for which data from the Sudan strain are available) versus 83% with Angola 2016 strains from the same region. Furthermore, the Uganda 2016 sequences corresponding to the NS genes NS3 and NS5 have the highest percentage identities (94% and 95%, respectively) with a Uganda 1948 strain relative to 85% and 84% with the Angola 2016 strains in the same regions. Together these BLAST analyses indicate that the Uganda 2016 YFV is most similar to strains in the East African genotype. Phylogenetic analyses confirm the BLAST analyses and place the Uganda 2016 YFV in a well-supported clade along with these East African genotype strains, whereas the Angola 2016 strains group with an Angola 1971 YFV (Figure), indicating that the Uganda outbreak in 2016 was not seeded by the Angola outbreak.

**Figure:**
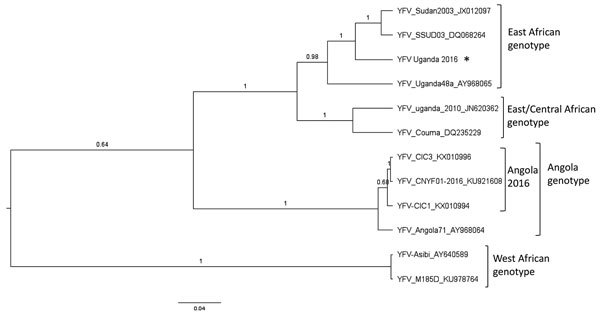
Bayesian maximum clade credibility tree of the Uganda 2016 YFV. Phylogenetic inference of the Uganda 2016 YFV strain (asterisk) representing partial coding regions of the membrane and envelope genes compared with reference YFV genotypes. Posterior probabilities are shown for each branch. Reference YFV strains are labeled with strain designation and GenBank accession numbers. YFV, yellow fever virus. Scale bar indicates nucleotide substitutions per site.

These findings reiterate the endemicity of YFV throughout the tropical regions of Africa because at least 2 concurrent yellow fever outbreaks of independent origins were identified in 2016. Our findings also highlight the importance of assessing the molecular epidemiology of the virus in outbreak investigations. These data improve our understanding of YFV epidemiology in Africa and support the previous studies of Mutebi and colleagues (*2*). In addition, removal of contaminating ribosomal RNA proved to be an effective method for unbiased enrichment of viral RNA in degraded samples to enhance sequencing sensitivity.
